# Surgical and Oncological Outcomes After Preoperative FOLFIRINOX Chemotherapy in Resected Pancreatic Cancer: An International Multicenter Cohort Study

**DOI:** 10.1245/s10434-022-12387-2

**Published:** 2022-12-20

**Authors:** Eran van Veldhuisen, Sjors Klompmaker, Quisette P. Janssen, Mohammed Abu Hilal, Adnan Alseidi, Alberto Balduzzi, Gianpaolo Balzano, Claudio Bassi, Frederik Berrevoet, Morgan Bonds, Olivier R. Busch, Giovanni Butturini, Kevin C. Conlon, Isabella M. Frigerio, Giuseppe K. Fusai, Johan Gagnière, Oonagh Griffin, Thilo Hackert, Asif Halimi, Tobias Keck, Jörg Kleeff, Ulla Klaiber, Knut J. Labori, Mickael Lesurtel, Giuseppe Malleo, Marco V. Marino, I. Quintus Molenaar, Michael B. Mortensen, Andrej Nikov, Michele Pagnanelli, Rupaly Pandé, Per Pfeiffer, Daniel Pietrasz, Elena Rangelova, Keith J. Roberts, Antonio Sa Cunha, Roberto Salvia, Oliver Strobel, Timo Tarvainen, Johanna W. Wilmink, Bas Groot Koerkamp, Marc G. Besselink, on behalf of the Scientific Committee of the European-African Hepato-Pancreato-Biliary Association, Alain Sauvanet, Lysiane Marthey, Lysiane Marthey, Christophe Laurent, Nicolas Régenet, Romain Coriat, Julien Taieb, Olivier Turini, Vincent Dubray, Raphael Bourdariat, Jean Baptiste Bachet, Lilian Schwartz

**Affiliations:** 1 Department of Surgery, Amsterdam UMC, University of Amsterdam, Cancer Center Amsterdam, Amsterdam, The Netherlands; 2 Department of Radiology, St. Antonius Hospital, Nieuwegein, The Netherlands; 3 Department of Surgery, Erasmus MC University Medical Center, Rotterdam, The Netherlands; 4 Department of Surgery, University Hospital Southampton NHS, Southampton, UK; 5 Department of General Surgery, Istituto Ospedaliero Fondazione Poliambulanza, Brescia, Italy; 6 Department of Surgery, University of California, San Francisco, USA; 7 Department of General and Pancreatic Surgery, The Pancreas Institute, University of Verona Hospital Trust, Verona, Italy; 8 Department of Surgery, Pancreas Unit, Ospedale San Raffaele, Milan, Italy; 9 Department of General and HPB Surgery, Gent University Hospital, Gent, Belgium; 10 HPB Surgery Unit, Pederzoli Hospital, Peschiera del Garda, Verona, Italy; 11 Department of Surgery, Trinity College Dublin and St. Vincent’s University Hospital, Dublin, Ireland; 12 HPB Surgery and Liver Transplantation Unit, Royal Free Hospital, London, UK; 13 Department of Digestive and Hepatobiliary Surgery-Liver Transplantation, University Hospital of Clermont-Ferrand, Clermont-Ferrand, France; 14 U1071 INSERM, Clermont-Auvergne University, Clermont-Ferrand, France; 15 Department of General, Visceral and Transplantation Surgery, Universitätsklinikum Heidelberg, Heidelberg, Germany; 16 Department of Surgery, Karolinska Institutet, Stockholm, Sweden; 17 Department of Surgery, Universitaet zu Luebeck, Luebeck, Germany; 18 Department of Visceral, Vascular and Endocrine Surgery, Martin Luther University Halle-Wittenberg, Halle, Germany; 19 Department of Hepato-Pancreato-Biliary Surgery, Oslo University Hospital, Oslo, Norway; 20 Department of Surgery and Liver Transplantation, Croix Rousse University Hospital, University of Lyon, Hospices Civils de LyonLyon, France; 21 Department of General Surgery, Azienda Ospedaliera, Ospedali Riuniti Villa Sofia-Cervello, Palermo, Italy; 22 Department of General Surgery, Hospital Universitario Marques de Valdecilla, Santander, Spain; 23 Department of Surgery, University Medical Center Utrecht, Utrecht, The Netherlands; 24 Department of Surgery, Odense Pancreas Center (OPAC), Odense University Hospital, Odense, Denmark; 25 Department of Surgery, 2nd Faculty of Medicine, Charles University and Central Military Hospital, Prague, Czech Republic; 26 Department of Surgery, University Hospital Birmingham, Birmingham, UK; 27 Department of Medical Oncology, Odense University Hospital, Odense, Denmark; 28 Department of Hepato-Biliary-Pancreatic Surgery, Liver Transplant Center, Paul Brousse Hospital, Université Paris-Sud, Université Paris-Saclay, Villejuif, France; 29 Department of Gastroenterological Surgery, Helsinki University Hospital, Helsinki, Finland; 30 Department of Medical Oncology, Amsterdam UMC, University of Amsterdam, Cancer Center Amsterdam, Amsterdam, The Netherlands

## Abstract

**Background:**

Preoperative FOLFIRINOX chemotherapy is increasingly administered to patients with borderline resectable (BRPC) and locally advanced pancreatic cancer (LAPC) to improve overall survival (OS). Multicenter studies reporting on the impact from the number of preoperative cycles and the use of adjuvant chemotherapy in relation to outcomes in this setting are lacking. This study aimed to assess the outcome of pancreatectomy after preoperative FOLFIRINOX, including predictors of OS.

**Methods:**

This international multicenter retrospective cohort study included patients from 31 centers in 19 European countries and the United States undergoing pancreatectomy after preoperative FOLFIRINOX chemotherapy (2012–2016). The primary end point was OS from diagnosis. Survival was assessed using Kaplan-Meier analysis and Cox regression.

**Results:**

The study included 423 patients who underwent pancreatectomy after a median of six (IQR 5–8) preoperative cycles of FOLFIRINOX. Postoperative major morbidity occurred for 88 (20.8%) patients and 90-day mortality for 12 (2.8%) patients. An R0 resection was achieved for 243 (57.4%) patients, and 259 (61.2%) patients received adjuvant chemotherapy. The median OS was 38 months (95% confidence interval [CI] 34–42 months) for BRPC and 33 months (95% CI 27–45 months) for LAPC. Overall survival was significantly associated with R0 resection (hazard ratio [HR] 1.63; 95% CI 1.20–2.20) and tumor differentiation (HR 1.43; 95% CI 1.08–1.91). Neither the number of preoperative chemotherapy cycles nor the use adjuvant chemotherapy was associated with OS.

**Conclusions:**

This international multicenter study found that pancreatectomy after FOLFIRINOX chemotherapy is associated with favorable outcomes for patients with BRPC and those with LAPC. Future studies should confirm that the number of neoadjuvant cycles and the use adjuvant chemotherapy have no relation to OS after resection.

**Supplementary Information:**

The online version contains supplementary material available at 10.1245/s10434-022-12387-2.

Pancreatic cancer is notorious for its poor prognosis.^1^ Based on the increasing incidence and lack of improvement in survival, pancreatic cancer is expected to become the second leading cause of cancer-related deaths worldwide in 2030.^2^ Resection combined with adjuvant chemotherapy has long been the current standard of care for pancreatic cancer.^3^

Many centers currently perform surgery for selected patients with locally advanced pancreatic cancer (LAPC) after several cycles of preoperative FOLFIRINOX chemotherapy comprising 5-fluorouracil, oxaliplatin, irinotecan, and folic acid. Previous studies reported a 5–33% resectability rate using this strategy, with median overall survival (OS) periods of 25 to 34 months.^4–6^ For patients with (borderline) resectable pancreatic cancer (BRPC), such a strategy also may be as effective as upfront surgery, with about 40% of patients not receiving adjuvant chemotherapy.^7^ As is already the case for other tumors,^8,9^ this has led to a shift toward preoperative treatment of patients with BRPC aimed at increasing the likelihood of a radical resection and hence improved survival.^10^

Two recent randomized trials from South Korea and the Netherlands provided evidence of the benefit of (gemcitabine-based) preoperative chemo(radio)therapy for patients with BRPC.^11,12^ Trials with preoperative FOLFIRINOX for BPRC are ongoing.

Most studies on the use of preoperative FOLFIRINOX for LAPC and BRPC are single-center studies that report little variation in perioperative strategies, such as the impact from the number of preoperative FOLFIRINOX cycles and the use of adjuvant therapy. Consequently, data on the impact that the number of preoperative and adjuvant cycles has on surgical outcomes and OS currently are lacking. We therefore performed a pan-European multicenter study to assess the surgical and oncologic outcomes of pancreatectomy after preoperative FOLFIRINOX chemotherapy aimed at identifying predictors of OS in order to further refine therapy, including details of preoperative and adjuvant therapy.

## Methods

This was a pan-European, retrospective, multicenter cohort study among centers represented by members of the European-African Hepato-Pancreato-Biliary Association (E-AHPBA). The study protocol, including an analysis framework, was approved by the E-AHPBA research and scientific committee and published online.^13^ All E-AHPBA members who performed pancreatectomy for pancreatic ductal adenocarcinoma (PDAC; further: pancreatic cancer) after preoperative FOLFIRINOX chemotherapy between 1 January 2012 and 31 December 2016 were invited to participate via e-mail. The institutional review board at the Amsterdam UMC (location: Academic Medical Center) waived the need for ethical review.

### Patients and Data Collection

All the participating surgeons completed an online survey (Google Survey, Mountain View, CA, USA) containing questions regarding standards of care and annual volume of pancreatic surgery in their center. Each center appointed a local study coordinator, who was responsible for questionnaire completion and data collection. Subsequently, all consecutive patients who underwent pancreatectomy (i.e., pancreatoduodenectomy, distal pancreatectomy, or total pancreatectomy for pathology-proven pancreatic cancer) after at least two cycles of preoperative FOLFIRINOX chemotherapy within the study period were retrieved. Each center submitted baseline data (sex, age, BMI, ASA classification, surgical history, and tumor characteristics), treatment data (number of preoperative and adjuvant therapy cycles, operative variables, adjuvant therapy), and outcome data (morbidity, mortality, hospital length of stay, histopathology, type of surgery, vascular resection, and survival) anonymously using predefined electronic case report forms (eCRF) data (Castor, Amsterdam, the Netherlands). The ethnicity of the included participants was not provided by the investigators.

All data were collected and analyzed by the central study coordinators (E.V. and S.K.). Patients were excluded if they had a non-pancreatic carcinoma diagnosis or essential missing staging or operative information (e.g., missing preoperative CT scan, operative reports, pathology reports), as previously defined by the study protocol.

### Definitions

Postoperative complications (morbidity) were scored and classified using the Clavien-Dindo classification of surgical complications.^14^ Major complications were defined as Clavien-Dindo grade 3a or higher. The definitions of the International Study Group on Pancreatic Surgery (ISGPS) were used to score postoperative pancreatic fistula,^15^ delayed gastric emptying,^16^ chyle leak^17^ and post-pancreatectomy hemorrhage.^18^ Ischemic morbidity was defined as an abdominal organ complication caused by surgery-related ischemia. Resection margins, including transection and circumferential margins, were categorized according to the Royal College of Pathologists’ definition and classified as R0 (margin to tumor ≥1 mm), R1 (margin to tumor <1 mm) or R2 (macroscopically positive margin).^19^ Preoperative resectability status was classified according to the National Comprehensive Cancer Networks (NCCN) Clinical Practice Guidelines for Pancreatic Adenocarcinoma version 2.2012 and categorized as LAPC (>180° arterial involvement or unreconstructible venous involvement), borderline resectable (<180° arterial involvement or reconstructible venous involvement), or primarily resectable (no arterial or venous involvement).^20^ Staging of disease was performed according to the eighth version of the American Joint Committee on Cancer (AJCC) tumor-node-metastasis (TNM) classification.^21^ Tumor response was defined according to the Response Evaluation Criteria in Solid Tumors (RECIST) 1.1 definitions and classified as complete response, partial response, stable disease, or progressive disease.^22^ Complications, re-admissions, and mortality all were recorded up to 90 days postoperatively. Overall survival, defined as the time between diagnosis and death, was based on last visit to the hospital, follow-up phone calls, or national security registries.

### Primary and Secondary End Points

The primary end point was OS, stratified by resectability status after preoperative FOLFIRINOX chemotherapy. The secondary outcomes were R0 resection margin (microscopically radical resection margin according to the Royal College of Pathologists definition),^19^ malignant lymph node ratio (LNR), response rates (i.e., RECIST), oncologic outcomes (i.e., progression-free survival, time to recurrence), and postoperative outcomes such as length of hospital stay, postoperative morbidity, and 90-day mortality. The analyses included the identification of practice variation (e.g., number of cycles, use of adjuvant chemotherapy) and the impact of this variation on surgical and oncologic outcome (e.g., differences in survival depending on the number of preoperative and adjuvant chemotherapy cycles) over time.

### Statistical Analysis

All statistical analyses were performed using STATA version 14.1 (StataCorp LP, College Station, TX, USA). Continuous data are presented as either mean ± standard deviation or median and interquartile range as appropriate, whereas categorical data are presented as frequencies and proportions. All confidence intervals (CIs) are 95%, and alpha levels for significance are lower than 0.05.

Multiple imputation was performed to correct for missing data. The primary analysis consisted of a multivariate Cox regression model based on backward stepwise elimination (*P* > 0.2) including all relevant patient characteristics (e.g., cycles of preoperative chemotherapy, resectability status at diagnosis, sex, age, adjuvant treatment) as covariates. All models were stratified for resectability status (BRPC vs LAPC).

Several sensitivity analyses were performed to further investigate practice variations and find potential targets for treatment improvement. These included the comparison of baseline characteristics and survival by number of FOLFIRINOX cycles, survival from date of surgery, time to recurrence, and correlation between preoperative and adjuvant chemotherapy. These analyses were repeated in the total cohort (i.e., before the exclusion of patients due to essential missing data) to reduce possible bias.

A final landmark analysis used a multivariable adjusted Cox model to test the association between adjuvant chemotherapy and survival starting at different time points to avoid immortal time bias (i.e., a patient must be alive to undergo treatment or experience an outcome). These time points were time of diagnosis, 3 months after surgery, and 8 months after surgery, excluding all patients receiving more than four cycles of preoperative FOLFIRINOX.

## Results

Of 56 initially responding centers, 29 centers across 18 European countries and 1 center in the United States fulfilled the eligibility criteria and included 552 patients who underwent pancreatectomy after preoperative FOLFIRINOX chemotherapy. Of the 29 participating centers, 5 centers had a median annual pancreatoduodenectomy volume of 20–40, 4 centers had a volume of 40–60, 8 centers had a volume of 60–80, and 12 centers performed more than 80 pancreatoduodenectomies annually. The median annual case volume of resections after FOLFIRINOX was 0–20 for 25 centers and 20–40 for 4 centers.

**Fig. 1:**
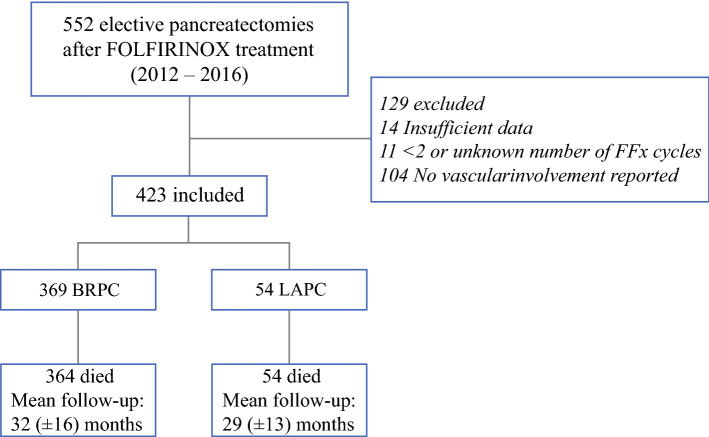
Study flow chart. BRPC, borderline resectable pancreatic cancer; LAPC, locally advanced pancreatic cancer; FFx, FOLFIRINOX; no., number

After excluding 11 patients who received fewer than two or an unknown number of FOLFIRINOX cycles, 14 patients who had missing essential data, and 104 patients because of missing details on vascular involvement (i.e., no differentiation between BRPC and LAPC), 423 patients remained eligible for this study (Fig. 1).

### Baseline Characteristics

The baseline characteristics of the 423 included patients are described in Table 1. Among the 423 patients, 369 (87.2%) received preoperative FOLFIRINOX chemotherapy for BRPC and 54 (12.8%) had preoperative FOLFIRINOX for LAPC. The patients received a median of 6 (IQR, 5–8) preoperative FOLFIRINOX cycles, and 126 (29.8%) patients received additional preoperative radiotherapy (stereotactic body radiation therapy for 96 patients [22.5%]). Dose-reductions of FOLFIRINOX were applied for 123 (29.1%) patients.

**Table 1:** Baseline characteristics

Baseline	BRPC		LAPC
	(*n* = 369) *n* (%)		(*n* = 54) *n* (%)
Mean age (years)	60.3 ± 9.5		61.1 ± 9.1
Median (IQR)	61.4 (54–67)		63.3 (55–68)
Female sex	171 (46.3)		25 (46.3)
Mean BMI (kg/m^2^)	24.7 ± 3.8		24.7 ± 5.8
Median (IQR)	24.3 (22–27)		23.8 (22–25)
Mean CCI	0.4 ± 0.6		0.3 ±0.4
Median (IQR)	0 (0–1)		0 (0–1)
Physical status			
ASA 1	85 (23.0)		13 (24.1)
ASA 2	222 (60.2)		35 (64.8)
ASA 3–4	58 (15.7)		6 (11.1)
ASA unknown	4 (1.1)		(.)
**Tumor characteristics after FOLFIRINOX**			
Mean tumor diameter (mm)	31 ± 10.4		37.9 ± 17.5
Median (IQR)	30 (24–37)		35 (27–46)
Tumor location			
Pancreas-head	273 (74.0)		31 (57.4)
Pancreas-body	55 (14.9)		18 (33.3)
Pancreas-tail	17 (4.6)		1 (1.9)
Periampullary	21 (5.7)		4 (7.4)
Multi-organ involvement	23 (6.3)		3 (5.7)
**Vascular involvement, any degree**			
Portomesenteric vein	300 (81.7)		40 (74.1)
Superior mesenteric artery	110 (29.9)		34 (63.0)
Celiac trunk	42 (11.4)		19 (35.2)
Hepatic artery	45 (12.2)		27 (50.0)
**Treatment characteristics**			
Mean preoperative no. of FOLFIRINOX cycles	6.7 ± 2.8		7.5 ± 4.1
Median (IQR)	6 (5–8)		6 (4–10)
Mean delta CA 19-9 (U/mL)	–1223.8 (4498.4)		–2633.9 (6611.3)
Median (IQR)	–132 (–636–11)		–490.5 (–1170–82)
Mean time to surgery (days)^a^	184.4 ± 96.1		216.5 ± 96.6
Median (IQR)	161.5 (116–231)		199 (147–253)
Procedure			
Pancreatoduodenectomy	293 (79.4)		28 (51.9)
Distal pancreatectomy	46 (12.5)		8 (14.8)
Total pancreatectomy or other	30 (8.1)		18 (33.3)

BRPC, borderline resectable pancreatic cancer; LAPC, locally advanced pancreatic cancer; IQR, interquartile range; BMI, body mass index; CCI, Charlson Cormorbidity Index; ASA, American Society of Anesthesiology

^a^From start of chemotherapy to date of surgery

At restaging, a complete radiologic response was observed for 14 (3.3%) patients, a partial response for 244 (57.7%) patients, stable disease in 157 (37.1%) patients, progression in 2 (0.5%) patients, and missing data on the RECIST response for 6 patients (1.4%).

After surgery, 259 (61.2%) patients received any type of adjuvant chemotherapy, and 51 (12.1%) patients received adjuvant radiotherapy. In an overview, Fig. S1 presents the number of administered (neo)adjuvant cycles per patient.

### Short-Term Outcomes

The most common surgical procedure was pancreatoduodenectomy (*n* = 321, 75.9%), followed by distal pancreatectomy (*n* = 54, 12.8%) and total pancreatectomy (*n* = 48, 11.3%). The most common surgical approach was open procedure (*n* = 405, 95.7%), whereas 18 (4.3%) patients underwent a minimally invasive resection (11 laparoscopic and 7 robot-assisted procedures). Venous resections, including wedge resections, were performed for 187 (44.2%) patients and major arterial resections for 38 (9%) patients. Of these procedures, 17 (4.0%) were common hepatic or proper hepatic artery resections, 15 (3.5%) were celiac axis resections, and 6 (1.4%) were superior mesenteric artery resections. Eight patients underwent other arterial resections (1.9%).

The mean hospital length of stay was 14 ± 11 days for BRPC and 18 ± 12 days for LAPC, with re-admission for 47 (13.2%) and 5 (9.4%) patients, respectively. An R0 resection was achieved for 243 (57.4%) patients (59.1% vs 46.3% respectively for BRPC and LAPC; *P* = 0.079). The median number of harvested lymph nodes was 21 (IQR, 15–33).

Postoperative major complications (i.e., Clavien-Dindo grade >3a) occurred for 88 (20.8%) patients. Data on morbidity were missing for five patients. Postoperative pancreatic fistula requiring re-intervention occurred for 10 patients (2.4%), post-pancreatectomy hemorrhage for 21 (5%) patients, and delayed gastric emptying for 27 (6.4%) patients. The postoperative 90-day mortality rate was 2.8% (12/423). Table 2 presents an overview of all the short-term outcomes.

**Table 2:** Secondary outcomes

Vascular resection	BRPC	LAPC	*P* Value
	(*n* = 369) *n* (%)	(*n* = 54) *n* (%)	
Venous			
Complete	102 (27.6)	29 (53.7)	<0.001
Wedge	50 (13.6)	6 (11.1)	0.83
Arterial			
Common/proper hepatic	10 (2.7)	7 (13.0)	0.003
Celiac trunk	5 (1.4)	10 (18.5)	<0.001
Superior mesenteric	4 (1.1)	2 (3.7)	0.171
Other (including accessory)	4 (1.1)	4 (7.4)	0.011
Pathology			
Tumor differentiation			
Well	52 (14.1)	8 (14.8)	0.836
Moderate	116 (31.4)	11 (20.4)	0.113
Poor/undifferentiated	66 (17.9)	2 (3.7)	0.005
Unknown	135 (36.6)	33 (61.1)	
Resection margin			
R0	218 (59.1)	25 (46.3)	0.079
R1	146 (39.6)	29 (53.7)	0.055
R2	4 (1.1)	( .)	>0.99
Unknown	1 (0.3)	( .)	
Mean malignant LNR (d)	0.1 ± 0.8	0.1 ± 0.1	0.484
Median (IQR)	0 (0–0)	0 (0–0)	
Distant metastasis	8 (2.2)	1 (1.9)	>0.99
90-Day outcomes			
Mean length of stay (days)	14.1 ± 11.4	17.9 ±12.1	0.026
Median (IQR)	12 (8–16)	14 (10–21)	
Complication grade			
0–2b (none or minor)	288 (78.0)	42 (77.8)	>0.99
3a (non-surgical)	23 (6.2)	4 (7.4)	0.764
3b (general anesthesia)	29 (7.9)	2 (3.7)	0.403
4a–4b (major)	13 (3.5)	5 (9.3)	0.065
5 (death)	11 (3.0)	1 (1.9)	>0.99
Unknown	5 (1.4)	( .)	
All cause 90-day mortality	11 (3.0)	1 (1.9)	>0.99
Adjuvant therapy			
Chemotherapy	233 (63.1)	26 (48.1)	0.037
Unknown	17 (4.6)	6 (11.1)	
Radiotherapy	47 (12.7)	4 (7.4)	0.37
Unknown	12 (3.3)	5 (9.3)	

Secondary outcomes between BRCP and LAPC, including significance testing.

BRPC, borderline resectable pancreatic cancer; LAPC, locally advanced pancreatic cancer; LNR, lymph node ratio; IQR, interquartile range

### Overall Survival and Predictors of Survival

**Fig. 2:**
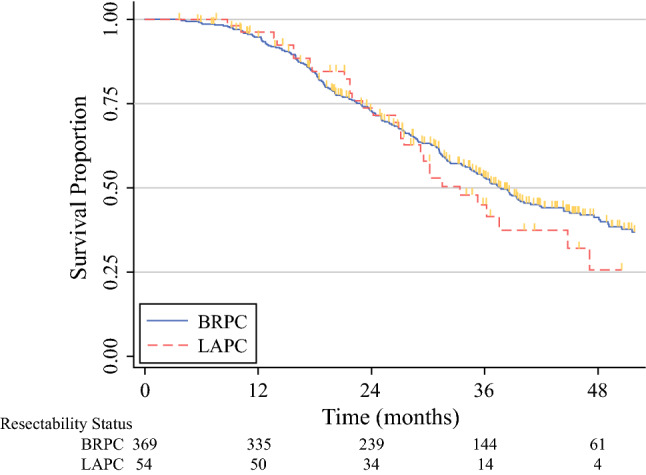
Overall survival for resected borderline resectable and locally advanced pancreatic cancer after preoperative FOLFIRINOX. Unadjusted Kaplan-Meier survival curves from the date of diagnosis, stratified by resectability status after preoperative FOLFIRINOX chemotherapy. The median survival was 38 months (95% CI 34–42 months) for BRPC and 33 months (95% CI 27–45 months) for LAPC (*P* = 0.490). BRPC, borderline resectable pancreatic cancer; LAPC, locally advanced pancreatic cancer

The median OS for the total cohort was 37 months (95% CI 34–40 months). After a mean follow-up period of 32 ± 16 months for BRPC and 29 ± 13 months for LAPC, the median OS was 38 months (95% CI 34–42 months) for BRPC and 33 months (95% CI 27–45 months) for LAPC (BRPC vs LAPC, *P* = 0.490; Fig. 2).

**Fig. 3:**
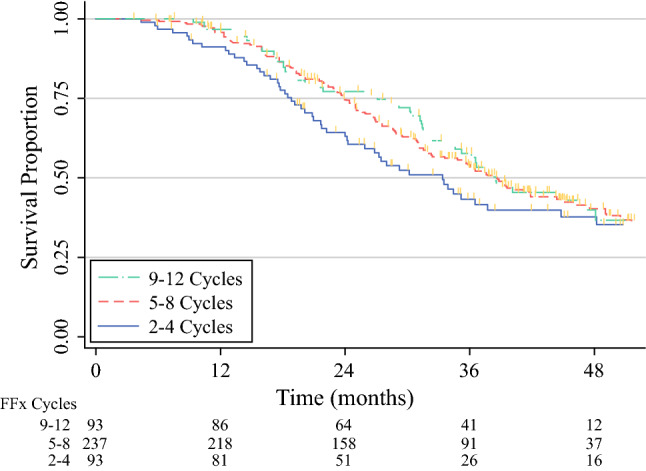
Overall survival stratified by the number of preoperative FOLFIRINOX cycles. Unadjusted Kaplan-Meier survival curves from the date of diagnosis, stratified by the number of preoperative FOLFIRINOX chemotherapy cycles. The median survival was 33 months (95% CI 24–45 months) for 2 to 4 cycles, 39 months (95% CI 32–45 months) for 5 to 8 cycles, and 39 months (95% CI 34–48 months) for 9 to 12 cycles (*P* = 0.335)

Likewise, no difference in survival was observed between the patients who received 2 to 4, 5 to 8, or 9 to 12 cycles of preoperative FOLFIRINOX. The median survival was 33 months (95% CI 24–45 months) for the patients who received 2 to 4 cycles, 39 months (95% CI 32–45 months) for those who received 5 to 8 cycles, and 39 months (95% CI 34–48 months) for those who received 9 to 12 cycles of preoperative FOLFIRINOX (*P* = 0.335; Fig. 3).

The lack of an association between the number of preoperative cycles and survival persisted when the analysis was performed for the total cohort of 527 patients (*P* = 0.0681) (i.e., before exclusion of patients due to essential missing data) (Fig. S1). The association of OS with the number of preoperative and adjuvant cycles also is depicted as a scatter plot in Fig. S1. The median survival from the date of surgery was 30 months (95% CI 27–34 months) for BRPC and 24 months (95% CI 17–32 months) for LAPC (*P* = 0.412; Fig. S3), which demonstrates survival from the date of surgery.

For the 387 patients (91.5%) who experienced disease recurrence (either local or distant), the median time to recurrence was 23 months (95% CI 22–26 months) for BRPC and 20 months (95% CI 16–25 months) for LAPC (*P* = 0.119; Fig. S4), which demonstrates time to recurrence.

In the univariable analysis (Fig. S5), the following factors were associated with OS: BMI, use of preoperative SBRT (stereotactic body radiation therapy), tumor diameter, celiac trunk involvement, tumor localization, malignant LNR, R0 resection, and tumor differentiation. However, only R0 resection (hazard ratio [HR], 1.63; 95% CI 1.20–2.20; *P* = 0.002) and tumor differentiation (HR 1.43; 95% CI 1.08–1.91; *P* = 0.017) remained significantly associated with OS in the multivariable analysis (Table 3). Neither BMI (HR 1.03; 95% CI 1.00–1.07; *P* = 0.067) nor malignant LNR (HR 1.11; 95% CI 0.99–1.26; *P* = 0.077) were statistically associated with OS, but were kept in the model for their clinical relevance. After the exclusion of 12 patients who died within 90 days after surgery, the analysis showed OS associated with tumor differentiation (HR 1.41; 95% CI 1.09–1.82; *P* = 0.01), malignant LNR (HR 1.16; 95% CI 1.03–1.30; *P* = 0.01), and R0 status (HR 1.63; 95% CI 1.26–2.27; *P* < 0.001) (Table 3).

**Table 3:** Factors associated with overall survival

Covariate	All patients (*n* = 423)				Excluding 90-day mortality (*n* = 411)			
	HR	*P* Value	LCI	UCI	HR	*P* Value	LCI	UCI
Age (years)	0.99	0.480	0.98	1.01	0.99	0.346	0.98	1.01
BMI, kg/m^2^	1.03	0.067	1.00	1.07	–	–	–	–
SBRT	0.76	0.121	0.53	1.08	–	–	–	–
Celiac trunk involvement	1.40	0.094	0.94	2.08	–	–	–	–
Tumor differentiation	1.43	0.017	1.08	1.91	1.41	0.01	1.09	1.82
Malignant LNR	1.11	0.077	0.99	1.26	1.16	0.01	1.03	1.30
R1 resection	1.63	0.002	1.20	2.20	1.69	<0.001	1.26	2.27

Multivariable Cox regression model, based on backward stepwise elimination (*P* > 0.1) after multiple imputation to include all potential significant predictors and confounders associated with survival. Age was included as a fixed covariate regardless of the model *P* value because of its obvious clinical relation to survival. The model was stratified by preoperative resectability status (BRPC vs LAPC) and based on imputation for missing information.

HR, hazard ratio; LCI, lower 95% confidence interval (CI); UCI, upper 95% CI; BMI, body-mass index; SBRT, (preoperative) stereotactic body radiation therapy; LNR, lymph node ratio: BRPC, borderline resectable pancreatic cancer; LAPC, locally advanced pancreatic cancer

### Sensitivity and Subgroup Analyses

Use of adjuvant chemotherapy was not associated with OS in the univariable screen (Fig. S5). When tested in a separate Cox model, including clinically relevant potential confounders (Fig. S6 demonstrates a Cox model including adjuvant chemotherapy), adjuvant chemotherapy remained unassociated with OS from the date of diagnosis (HR 0.91; 95% CI 0.68–1.20; *P* = 0.496).

Two additional landmark analyses starting respectively 3 and 8 months after surgery confirmed this lack of association (Fig. S6 demonstrates two landmark analyses including adjuvant chemotherapy). Even when patients who received six or more adjuvant cycles (HR 0.87; 95% CI 0.56–1.36; *P* = 0.539) and those who received only two to four preoperative cycles of FOLFIRINOX (HR 1.20; 95% CI 0.45–3.20; *P* = 0.715) were assessed separately, adjuvant chemotherapy remained unassociated with OS (Fig. S6 demonstrates the sensitivity analyses). Baseline characteristics stratified by number of preoperative cycles are demonstrated in Fig. S7.

In addition, a sensitivity analysis was performed to investigate the impact of preoperative cycles stratified by a duration of <5 months versus ≥5 months of preoperative therapy respectively. When survival was compared between the patients who received <5 months of preoperative therapy and those who received ≥5 months of preoperative therapy, no difference in survival was found. The median survival for the patients who received <5 months of preoperative therapy was 33 months (95% CI 24–45 months) compared with 39 months (95% CI 35–44 months) for the patients who received ≥5 months of preoperative therapy. Likewise, a Cox model including relevant clinical factors and duration of preoperative therapy demonstrated no association between duration of preoperative therapy and OS (*P* = 0.233; Fig. S8).

## Discussion

This pan-European multicenter cohort study of 423 patients undergoing pancreatic resection after preoperative FOLFIRINOX found a 90-day mortality of 2.8% and a median OS of 38 months for the patients with BRPC and 33 months for those with LAPC. Somewhat unexpected, the number of preoperative FOLFIRINOX cycles and the use of adjuvant chemotherapy were not related to OS in this setting. Likewise, the use of preoperative radiotherapy was not independently associated with OS. These findings require confirmation in prospective studies.

Since the demonstration of FOLFIRINOX superiority over gemcitabine for metastatic pancreatic cancer,^23^ this regimen is increasingly used for patients with BRPC and those with LAPC. A meta-analysis including 315 patients with LAPC demonstrated a resectability rate of 26% and an R0 resection rate of 78% (95% CI 60–92%) after preoperative FOLFIRINOX treatment.^5^ Although the R0 resection rate in our study was somewhat lower (57%), it confirmed that radical tumor resection remains an important predictor of OS. However, the considerable heterogeneity between the included studies and the substantial proportion of missing data in the systematic review prevent a reliable comparison.^5^

The impact of the number of preoperative FOLFIRINOX cycles in pancreatic cancer also has been addressed in previous studies. Truty et al.^24^ reported that receiving at least six cycles of chemotherapy was a favorable prognostic factor for survival after resection of BPRC/LAPC. The results in the current study are in accordance with those of a previous systematic review of 313 patients who had BRPC treated with FOLFIRINOX followed by resection.^10^ Similar to our findings, this study reported no association between the number of neoadjuvant cycles and OS. Based on the retrospective design of the current study, we cannot rule out the possibility that the patients with more aggressive tumor biology may have received a longer duration of preoperative chemotherapy, or that the patients with a good response to preoperative therapy proceeded to surgery early because of a favorable response to FOLFIRINOX. In addition, the current study may not have been able to demonstrate an association between the number of FOLFIRINOX cycles and OS due to a lack of power (i.e., sample that was too small) because the study was not powered to demonstrate this association. Future prospective studies with a predefined treatment protocol are needed to confirm this hypothesis.

Adjuvant chemotherapy was not associated with OS in the current study. However, as demonstrated in a previously published sub-analysis of the current cohort, the use of adjuvant therapy was associated with improved survival for patients with node-positive disease.^25^ These patients who were treated with adjuvant therapy demonstrated a 26-month overall survival period compared with 13 months for the patients who did not receive adjuvant treatment (*P* = 0.004).^25^ This emphasizes the need for personalized treatment of patients with pancreatic cancer (i.e., identifying clinical factors that might predict treatment efficacy) to prevent futile treatment.

Another recently published single-center study assessed the impact of adjuvant chemotherapy after preoperative chemotherapy on 245 patients with resected pancreatic cancer. The authors reported that adjuvant therapy was marginally associated with OS despite poor prognostic factors in these patients.^26^ These results are comparable with those of a recent study demonstrating that patients with a poor response to neoadjuvant treatment, defined as no normalization of CA 19-9 after neoadjuvant therapy, demonstrated a positive impact of adjuvant treatment on survival.^27^ An explanation of why the use of adjuvant therapy was not associated with survival in the current study is lacking. In other cancer types, such as breast and rectal cancer, some evidence exists to suggest that patients with a good response to neoadjuvant treatment may not benefit from adjuvant therapy.^28,29^ Possibly, a good overall response to neoadjuvant FOLFIRINOX treatment in the current cohort could explain these outcomes. Future studies should aim to identify subgroups of patients who benefit from adjuvant therapy after preoperative FOLFIRINOX chemotherapy.

It is interesting to assess our findings in light of ongoing randomized trials. For example, the ongoing Dutch multicenter PREOPANC-2 trial uses eight cycles of neoadjuvant FOLFIRINOX without adjuvant treatment as the intervention arm of the study.^30^ Moreover, the multicenter NorPACT-1 trial in the Nordic countries investigates the added value of neoadjuvant FOLFIRINOX followed by adjuvant chemotherapy for patients with (borderline) resectable pancreatic cancer.^31^ Finally, the NEOLAP trial in Germany compares preoperative FOLFIRINOX with gemcitabine-abraxane in patients with LAPC to determine the optimal preoperative treatment regimen for LAPC.^32^ In both the NorPACT-1 and NEOLAP trials, all the patients are advised to use adjuvant treatment after neoadjuvant chemotherapy. Only the PREOPANC-2 trial does not use adjuvant treatment after neoadjuvant FOLFIRINOX. Comparing the results of these trials with the findings of the current study may provide more evidence on the added value of adjuvant chemotherapy after initial preoperative treatment with regard to survival.

The results of the current study should be interpreted in light of some limitations. First, this study included only patients who underwent pancreatectomy after preoperative FOLFIRINOX. Patients who did not undergo a resection or who had upfront resection were not included. Therefore, no conclusion on the efficacy of FOLFIRINOX per se can be provided by this study. Such evidence should come from the previously mentioned randomized trials.

Second, as a result of the retrospective and non-randomized study design, we cannot rule out selection or reporting bias through self-selection of the participating centers. This may be reflected by the relatively low morbidity rate in this study and the relatively low number of annual cases contributed per center. Yet, anonymity of the participating centers, a predefined study protocol, and the fact that 60% of patients were retrieved from prospectively maintained databases reduced the risk of such biases. In addition, because a substantial amount of data on vascular involvement was missing, it was not possible to determine the resectability status of all the patients. These patients were excluded from the primary analysis, which could have introduced selection bias. However, when repeating the analysis of the total cohort, we found that the results did not change.

Third, the treatment standards and resectability criteria after FOLFIRINOX chemotherapy may have varied between centers.

Fourth, we obtained imaging reports only after FOLFIRINOX treatment, so we could not differentiate between LAPC and BRPC at the time of diagnosis. Instead, we used resectability status at the time of resection, preventing generalizability of this study’s results to patients at first presentation.

The strengths of the current study were the multicenter, international study design, which allowed for the identification of practice variation and its lack of impact on OS. Based on these results, we identified several predictors for survival. These did not include the duration of preoperative treatment or the use of adjuvant treatment.

## Conclusion

In conclusion, the current study demonstrated an acceptable morbidity and mortality after pancreatectomy following preoperative FOLFIRINOX chemotherapy, with a promising 38 months of OS for BRPC and 33 months for LAPC. We found no evidence to support the hypothesis that a higher number of preoperative cycles of FOLFIRINOX or adjuvant chemotherapy is associated with prolonged OS in this setting. Future prospective and randomized studies should determine the optimal duration of preoperative treatment and assess the true impact of adjuvant chemotherapy after neoadjuvant FOLFIRINOX on subgroups of interest.

## Supplementary Information

Below is the link to the electronic supplementary material.

Supplementary file 1 (DOCX 134 KB)

Supplementary file 2 (DOCX 205 KB)

Supplementary file 3 (DOCX 119 KB)

Supplementary file 4 (DOCX 116 KB)

Supplementary file 5 (DOCX 15 KB)

Supplementary file 6 (DOCX 21 KB)

Supplementary file 7 (DOCX 16 KB)

Supplementary file 8 (DOCX 16 KB)
