# Supplementary material for: Surgical and Oncological Outcomes After Preoperative FOLFIRINOX Chemotherapy in Resected Pancreatic Cancer: An International Multicenter Cohort Study
Source: Ann Surg Oncol. 2022 Dec 20;30(3):1463–73. doi: 10.1245/s10434-022-12387-2 (PMC9908650; doi:10.1245/s10434-022-12387-2)
Supplement: Supplementary file 1 — (DOCX 134 KB) [file 10434_2022_12387_MOESM1_ESM.docx]

SUPPLEMENT S1. OVERALL SURVIVAL STRATIFIED BY NUMBER OF CYCLES OF PREOPERATIVE FOLFIRINOX IN THE TOTAL COHORT BEFORE EXCLUSION


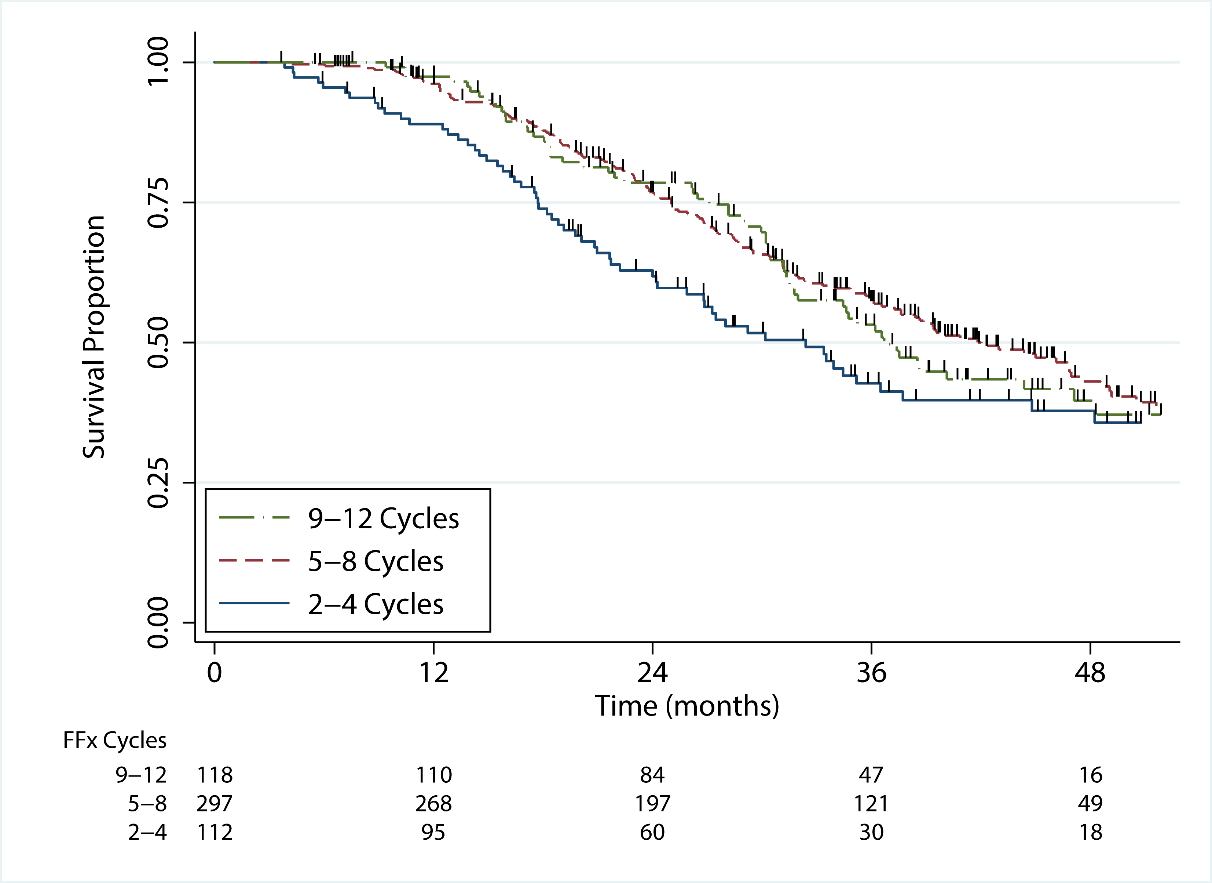


CAPTION: Unadjusted Kaplan-Meier survival curves from date of diagnosis, stratified by number of cycles of preoperative FOLFIRINOX chemotherapy in the total cohort (n=527) before exclusion due to missing data on vascular involvement. Median survival was 32 (95% CI 24-38) months for 2-4 preoperative cycles, 42 (95% CI 37-48) months for 5-8 cycles and 37 (95% CI 31-47) months for 9-12 cycles (P=0.068).
