# Supplementary material for: Surgical and Oncological Outcomes After Preoperative FOLFIRINOX Chemotherapy in Resected Pancreatic Cancer: An International Multicenter Cohort Study
Source: Ann Surg Oncol. 2022 Dec 20;30(3):1463–73. doi: 10.1245/s10434-022-12387-2 (PMC9908650; doi:10.1245/s10434-022-12387-2)
Supplement: Supplementary file 2 — (DOCX 205 KB) [file 10434_2022_12387_MOESM2_ESM.docx]

SUPPLEMENTAL DIGITAL CONTENT 2. SCATTER PLOT OF PREOPERATIVE CYCLES OF FOLFIRINOX AND ADJUVANT CHEMOTHERAPY

A.


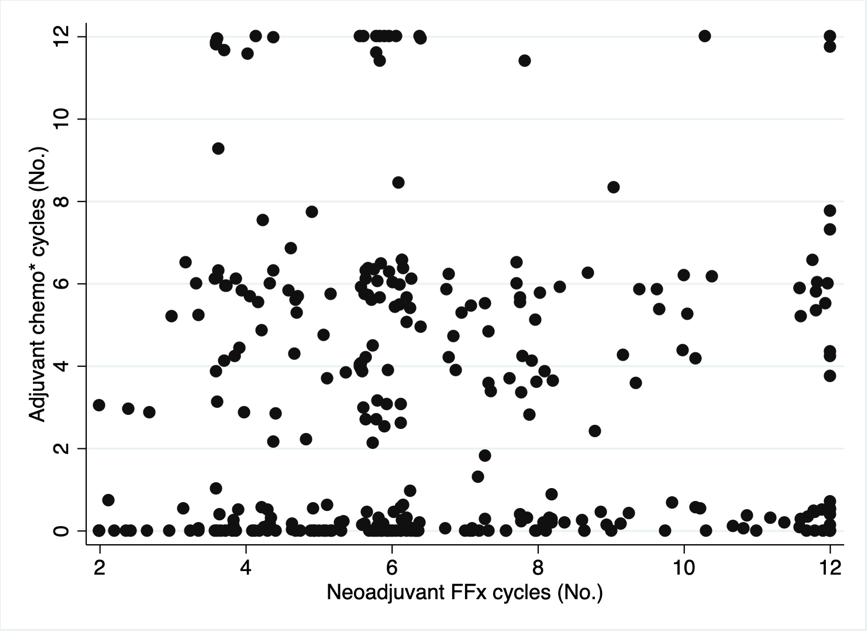


B.


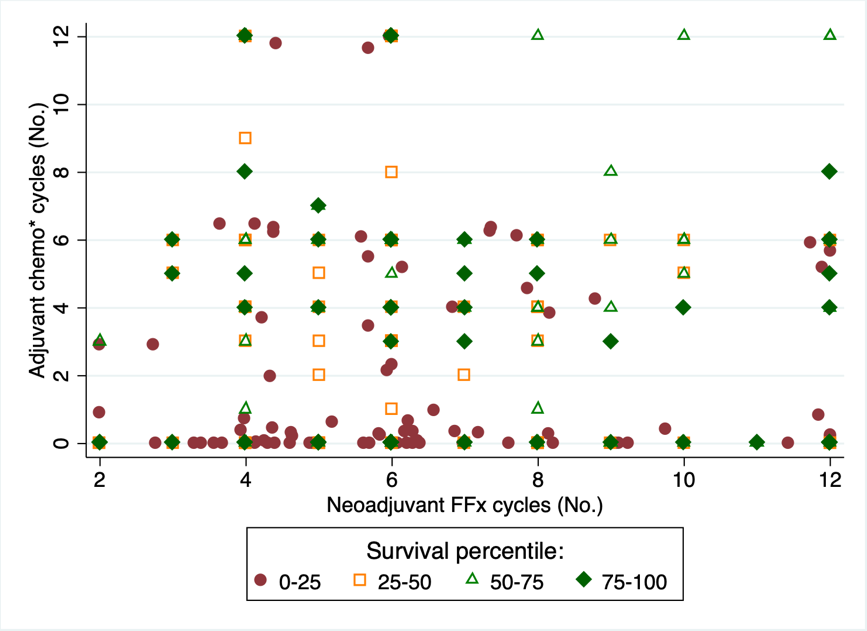


CAPTION: Scatter plot of neoadjuvant and adjuvant chemotherapy cycles for (A) individual patients included in the study and (B) grouped by survival percentile. The number of chemotherapy cycles were truncated at 12. Abbreviations: FFx, FOLFIRINOX; No., number. *Any chemotherapy regimen, including non-FOLFIRINOX agents.
