# Supplementary material for: Surgical and Oncological Outcomes After Preoperative FOLFIRINOX Chemotherapy in Resected Pancreatic Cancer: An International Multicenter Cohort Study
Source: Ann Surg Oncol. 2022 Dec 20;30(3):1463–73. doi: 10.1245/s10434-022-12387-2 (PMC9908650; doi:10.1245/s10434-022-12387-2)
Supplement: Supplementary file 3 — (DOCX 119 KB) [file 10434_2022_12387_MOESM3_ESM.docx]

SUPPLEMENTAL DIGITAL CONTENT 3. SURVIVAL FROM DATE OF SURGERY


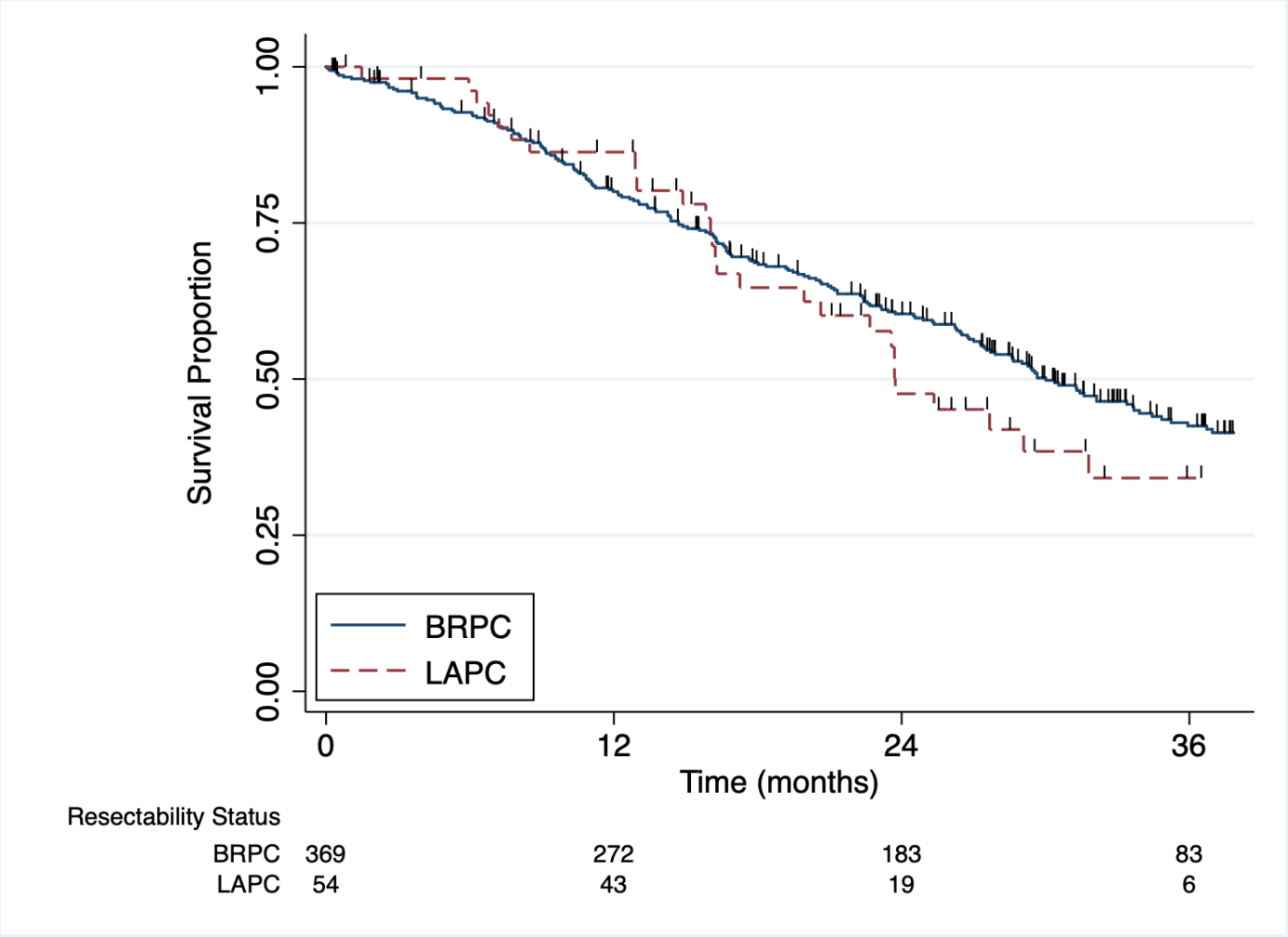


CAPTION: Unadjusted Kaplan-Meier survival curves from date of surgery, stratified by resectability status after preoperative FOLFIRINOX chemotherapy. Median survival was 30 (95%CI: 27-34) months for BRPC and 24 (95%CI: 17-32) months for LAPC (P = 0.412). Abbreviations: BRPC, borderline resectable pancreatic cancer; LAPC, locally advanced pancreatic cancer.
