# Supplementary material for: Surgical and Oncological Outcomes After Preoperative FOLFIRINOX Chemotherapy in Resected Pancreatic Cancer: An International Multicenter Cohort Study
Source: Ann Surg Oncol. 2022 Dec 20;30(3):1463–73. doi: 10.1245/s10434-022-12387-2 (PMC9908650; doi:10.1245/s10434-022-12387-2)
Supplement: Supplementary file 4 — (DOCX 116 KB) [file 10434_2022_12387_MOESM4_ESM.docx]

SUPPLEMENTAL DIGITAL CONTENT 4. TIME TO RECURRENCE


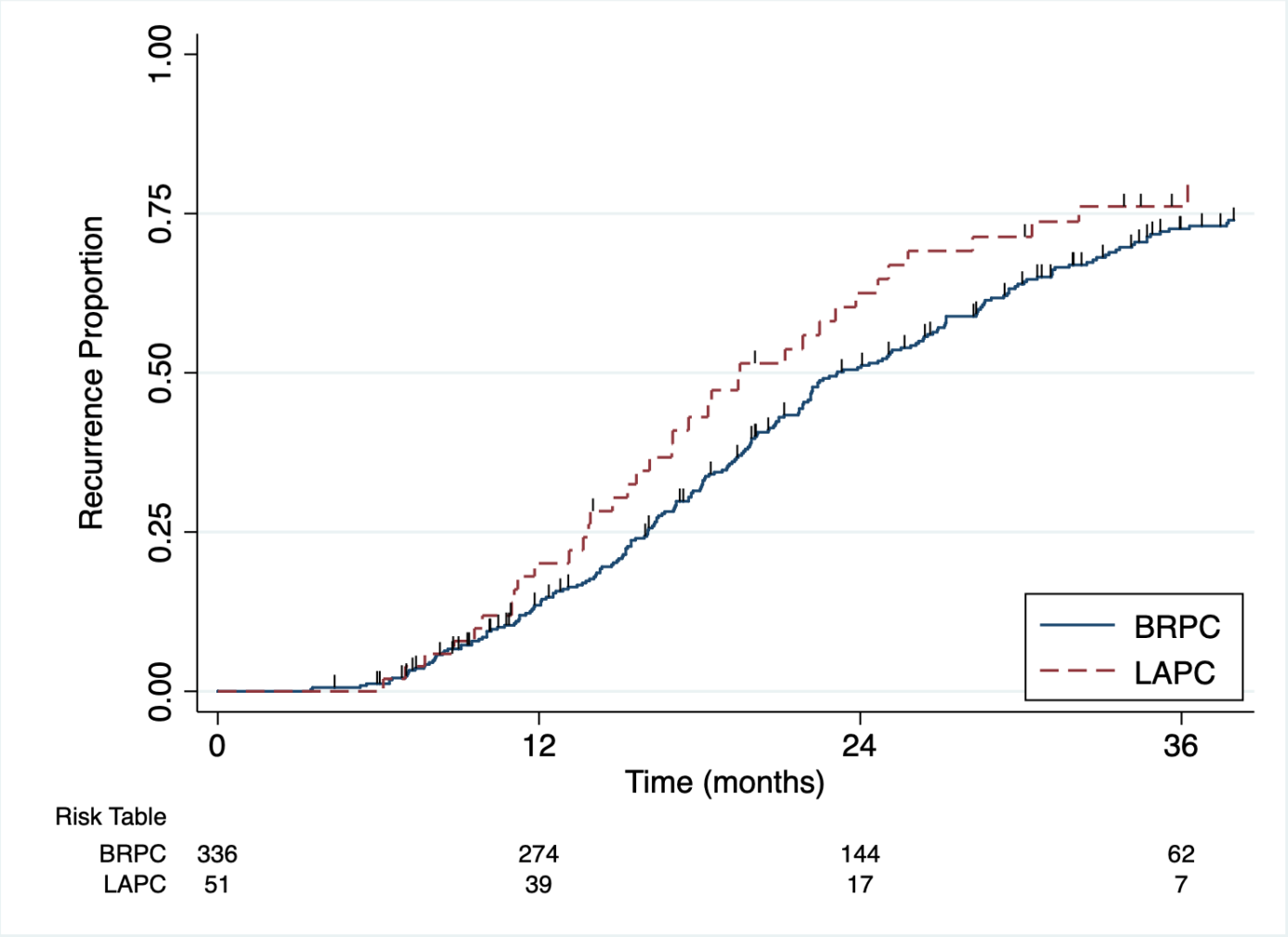


CAPTION: Time to recurrence, either local or distant, in 387 patients with a recorded recurrence event status. Median time to recurrence was 23 (95%CI: 22-26) months for BRPC and 20 (95%CI: 16-25) months for LAPC (P = 0.119). Abbreviations: BRPC, borderline resectable pancreatic cancer; LAPC, locally-advanced pancreatic cancer.
