# Supplementary material for: Surgical and Oncological Outcomes After Preoperative FOLFIRINOX Chemotherapy in Resected Pancreatic Cancer: An International Multicenter Cohort Study
Source: Ann Surg Oncol. 2022 Dec 20;30(3):1463–73. doi: 10.1245/s10434-022-12387-2 (PMC9908650; doi:10.1245/s10434-022-12387-2)
Supplement: Supplementary file 5 — (DOCX 15 KB) [file 10434_2022_12387_MOESM5_ESM.docx]

SUPPLEMENTAL DIGITAL CONTENT 5. UNIVARIATE SCREEN

| **Variable** | **HR** | **P-value** | **LCI** | **UCI** |
| --- | --- | --- | --- | --- |
| Age | 1.00 | 0.914 | 0.99 | 1.01 |
| Female sex | 1.03 | 0.798 | 0.80 | 1.34 |
| BMI, kg/cm2 | 1.03 | 0.081 | 1.00 | 1.07 |
| Charlson Comorbidity Index | 1.17 | 0.170 | 0.93 | 1.47 |
| **Physical Status** |  |  |  |  |
| ASA-1 | 0.82 | 0.200 | 0.60 | 1.11 |
| ASA-2 | 1.08 | 0.566 | 0.83 | 1.41 |
| ASA-3/4 | 1.19 | 0.346 | 0.83 | 1.71 |
| ASA Unknown | 0.79 | 0.739 | 0.20 | 3.18 |
| **Tumor location** |  |  |  |  |
| Pancreas-Head | 0.69 | 0.009 | 0.52 | 0.91 |
| Pancreas-Body | 1.29 | 0.132 | 0.93 | 1.81 |
| Pancreas-Tail | 1.08 | 0.807 | 0.59 | 1.98 |
| Periampullary | 1.56 | 0.080 | 0.95 | 2.55 |
| Multi-organ involvement | 1.29 | 0.309 | 0.79 | 2.12 |
| Tumor diameter, mm | 1.01 | 0.090 | 1.00 | 1.02 |
| **Vascular involvement** |  |  |  |  |
| PV-SMV | 0.98 | 0.893 | 0.70 | 1.36 |
| SMA | 0.96 | 0.750 | 0.73 | 1.26 |
| Celiac trunk | 1.58 | 0.011 | 1.11 | 2.25 |
| Hepatic artery | 1.24 | 0.204 | 0.89 | 1.73 |
| **Induction treatment** |  |  |  |  |
| Cycles of FFx | 0.98 | 0.286 | 0.93 | 1.02 |
| SBRT | 0.62 | 0.004 | 0.44 | 0.86 |
| Delta CA 19-9, U/mL | 1.00 | 0.558 | 1.00 | 1.00 |
| Time to surgery, d | 1.00 | 0.285 | 1.00 | 1.00 |
| **Type of resection** |  |  |  |  |
| Pancreatoduodenectomy | 0.66 | 0.005 | 0.49 | 0.88 |
| Distal pancreatectomy | 1.46 | 0.044 | 1.01 | 2.10 |
| Total pancreatectomy or other | 1.42 | 0.081 | 0.96 | 2.12 |
| **Postoperative factors** |  |  |  |  |
| Tumor differentiation | 1.63 | <0.001 | 1.29 | 2.05 |
| Resection margin (R1/R2) | 1.91 | <0.001 | 1.47 | 2.49 |
| Malignant LNR, mean (SD), d | 1.20 | 0.001 | 1.08 | 1.34 |
| Adjuvant chemotherapy | 0.93 | 0.594 | 0.71 | 1.21 |
|  |  |  |  |  |

CAPTION: unadjusted screen of factors to predict overall survival. Abbreviations: ASA, American Society of Anesthesiologists; BMI, body-mass index; PV – SMV, portal vein and superior mesenteric vein; SMA, superior mesenteric artery; FFx, FOLFIRINOX; SBRT, stereotactic body radiation therapy; d, days; LNR, lymph node ratio; SD, standard deviation; HR, hazard ratio; LCI, lower 95% confidence interval; UCI, upper 95% confidence interval.
