# Supplementary material for: Surgical and Oncological Outcomes After Preoperative FOLFIRINOX Chemotherapy in Resected Pancreatic Cancer: An International Multicenter Cohort Study
Source: Ann Surg Oncol. 2022 Dec 20;30(3):1463–73. doi: 10.1245/s10434-022-12387-2 (PMC9908650; doi:10.1245/s10434-022-12387-2)
Supplement: Supplementary file 6 — (DOCX 21 KB) [file 10434_2022_12387_MOESM6_ESM.docx]

SUPPLEMENTAL DIGITAL CONTENT 6. BASELINE CHARACTERISTICS STRATIFIED BY NUMBER OF PREOPERATIVE CYCLES OF FOLFIRINOX

|  | **2-4 Cycles** | | **5-8 Cycles** | | **9-12 Cycles** | | **P-value*** | |
| --- | --- | --- | --- | --- | --- | --- | --- | --- |
| **Baseline** | **n=93** |  | **n=237** |  | **n=93** |  |  |  |
| Age, mean (SD), y | 60.6 | (9.5) | 60.4 | (9.6) | 60.3 | (8.9) | 0.824 | |
| Median (IQR) | 61.4 | (55-68) | 61.6 | (54-67) | 61.1 | (54-67) |  | |
| Female sex, No. (%) | 43 | (46.2) | 109 | (46.0) | 44 | (47.3) | 0.991 | |
| BMI, mean (SD), y | 24.4 | (3.4) | 24.9 | (4.6) | 24.1 | (3.5) | 0.700 | |
| Median (IQR) | 23.9 | (22-27) | 24.5 | (22-27) | 24.3 | (21-26) |  | |
| Charlson CI, mean (SD) | 0.4 | (0.6) | 0.4 | (0.7) | 0.3 | (0.5) | 0.216 | |
| Median (IQR) | 0 | (0-1) | 0 | (0-1) | 0 | (0-1) |  | |
| Physical Status, No. (%) |  |  |  |  |  |  |  | |
| ASA-1 | 17 | (18.3) | 60 | (25.3) | 21 | (22.6) | 0.409 | |
| ASA-2 | 65 | (69.9) | 135 | (57.0) | 57 | (61.3) | 0.095 | |
| ASA-3/4 | 10 | (10.8) | 39 | (16.5) | 15 | (16.1) | 0.436 | |
| ASA Unknown | 1 | (1.1) | 3 | (1.3) |  | ( .) | 0.816 | |
| **Tumor characteristics** |  |  |  |  |  |  |  | |
| Tumor diameter, mean (SD), mm | 32.5 | (13.8) | 31.5 | (10.3) | 32.2 | (13.1) | 0.852 | |
| Median (IQR) | 31 | (24-39) | 30 | (25-37) | 30 | (22-40) |  | |
| Tumor location, No. (%) |  |  |  |  |  |  |  | |
| Pancreas-Head | 68 | (73.1) | 172 | (72.6) | 64 | (68.8) | 0.772 | |
| Pancreas-Body | 15 | (16.1) | 42 | (17.7) | 16 | (17.2) | 0.984 | |
| Pancreas-Tail | 3 | (3.2) | 8 | (3.4) | 7 | (7.5) | 0.275 | |
| Periampullary | 6 | (6.5) | 15 | (6.3) | 4 | (4.3) | 0.851 | |
| Multi-organ involvement, No. (%) | 8 | (8.7) | 9 | (3.8) | 9 | (9.7) | 0.068 | |
| Vascular involvement, No. (%) |  |  |  |  |  |  |  | |
| Portomesenteric vein | 79 | (84.9) | 191 | (81.3) | 70 | (75.3) | 0.247 | |
| Superior mesenteric artery | 38 | (40.9) | 69 | (29.2) | 37 | (39.8) | 0.057 | |
| Celiac trunk | 18 | (19.4) | 27 | (11.4) | 16 | (17.2) | 0.125 | |
| Hepatic artery | 18 | (19.4) | 42 | (17.8) | 12 | (12.9) | 0.455 | |
| **Treatment characteristics** |  |  |  |  |  |  |  | |
| Number of preoperative cycles FOLFIRINOX, mean (SD), No. | 3.6 | (0.7) | 6.3 | (0.9) | 11.5 | (2.5) | - | |
| Median (IQR) | 4 | (3-4) | 6 | (6-7) | 11 | (10-12) |  | |
| Delta CA 19-9, mean (SD), U/mL | -305.8 | (528.6) | -1468 | (5007.0) | -2247.6 | (6238.6) | 0.020 | |
| Median (IQR) | -67 | (-351--21) | -161 | (-705--6) | -346 | (-2005--66) |  | |
| Time to surgery^±^, mean (SD), d | 146.7 | (85.0) | 181.6 | (85.7) | 250.8 | (105.0) | <0.01 | |
| Median (IQR) | 115 | (85-194) | 153.5 | (121-217) | 229 | (181-271) |  | |
| Procedure, No. (%) |  |  |  |  |  |  |  | |
| Pancreatoduodenectomy | 72 | (77.4) | 186 | (78.5) | 63 | (67.7) | 0.125 | |
| Distal pancreatectomy | 10 | (10.8) | 29 | (12.2) | 15 | (16.1) | 0.5 | |
| Total pancreatectomy or other | 11 | (11.8) | 22 | (9.3) | 15 | (16.1) | 0.205 | |

CAPTION: Abbreviations: # Missing data; ± Start of chemo to date of surgery; * Based on univariable logistic regression.
