# Supplementary material for: Surgical and Oncological Outcomes After Preoperative FOLFIRINOX Chemotherapy in Resected Pancreatic Cancer: An International Multicenter Cohort Study
Source: Ann Surg Oncol. 2022 Dec 20;30(3):1463–73. doi: 10.1245/s10434-022-12387-2 (PMC9908650; doi:10.1245/s10434-022-12387-2)
Supplement: Supplementary file 7 — (DOCX 16 KB) [file 10434_2022_12387_MOESM7_ESM.docx]

SUPPLEMENTAL DIGITAL CONTENT 7. THE ASSOCIATION BETWEEN ADJUVANT CHEMOTHERAPY AND SURVIVAL

| A. | **All patients starting at time of diagnosis (n=423)** | | | | | |
| --- | --- | --- | --- | --- | --- | --- |
| **Covariate** | **HR** | **P-Value** | **LCI** | **UCI** | |  |
| Number of FOLFIRINOX cycles | 0.96 | 0.086 | 0.92 | 1.01 | |  |
| Age per year | 1.00 | 0.626 | 0.98 | 1.01 | |  |
| Tumor diameter per cm | 1.01 | 0.276 | 0.99 | 1.02 | |  |
| Malignant lymph-node-ratio | 1.18 | 0.005 | 1.05 | 1.32 | |  |
| Tumor differentiation per grade | 1.52 | 0.001 | 1.21 | 1.92 | |  |
| Relative CA19-9 response per % | 0.91 | 0.429 | 0.70 | 1.17 | |  |
| Adjuvant chemotherapy versus none | 0.91 | 0.496 | 0.68 | 1.20 | |  |
| B. | **Starting at 3 months follow-up after surgery (n=392)** | | | | | |
| **Covariate** | **HR** | **P-Value** | **LCI** | **UCI** | |  |
| Number of FOLFIRINOX cycles | 0.99 | 0.680 | 0.94 | 1.04 | |  |
| Age per year | 1.00 | 0.639 | 0.98 | 1.01 | |  |
| Tumor diameter per cm | 1.00 | 0.486 | 0.99 | 1.02 | |  |
| Malignant lymph-node-ratio | 1.20 | 0.002 | 1.07 | 1.34 | |  |
| Tumor differentiation per grade | 1.54 | 0.001 | 1.20 | 1.97 | |  |
| Relative CA19-9 response per % | 0.89 | 0.373 | 0.68 | 1.16 | |  |
| Adjuvant chemotherapy versus none | 0.99 | 0.947 | 0.73 | 1.34 | |  |
| C. | **Starting at 8 months follow-up after surgery (n=257)*** | | | | | |
| **Covariate** | **HR** | **P-Value** | **LCI** | **UCI** | |  |
| Number of FOLFIRINOX cycles | 0.96 | 0.186 | 0.90 | 1.02 | |  |
| Age per year | 1.00 | 0.938 | 0.98 | 1.02 | |  |
| Tumor diameter per cm | 1.01 | 0.227 | 0.99 | 1.02 | |  |
| Malignant lymph-node-ratio | 1.27 | 0.001 | 1.11 | 1.47 | |  |
| Tumor differentiation per grade | 1.53 | 0.007 | 1.13 | 2.07 | |  |
| Relative CA19-9 response | 0.82 | 0.271 | 0.57 | 1.18 | |  |
| Adjuvant chemotherapy |  | | | |  |  |
| None | referent |  |  |  | |  |
| 1-5 cycles | 1.28 | 0.246 | 0.84 | 1.96 | |  |
| 6 cycles or more | 0.87 | 0.539 | 0.56 | 1.36 | |  |
| D. | **Starting at 8 months follow-up and excluding patients receiving >4 cycles of** preoperative **FOLFIRINOX chemotherapy (n=57)**** | | | | | |
| **Covariate** | **HR** | **P-Value** | **LCI** | **UCI** | |  |
| Age per year | 1.02 | 0.390 | 0.97 | 1.07 | |  |
| Tumor diameter per cm | 1.02 | 0.074 | 1.00 | 1.05 | |  |
| Malignant lymph-node-ratio | 11.48 | 0.055 | 0.94 | 139.51 | |  |
| Tumor differentiation per grade | 0.90 | 0.831 | 0.34 | 2.38 | |  |
| Relative CA19-9 response | 1.82 | 0.084 | 0.92 | 3.59 | |  |
| Adjuvant chemotherapy |  |  |  |  | |  |
| None | referent |  |  |  | |  |
| 1-5 cycles | 1.63 | 0.319 | 0.62 | 4.24 | |  |
| 6 cycles or more | 1.20 | 0.715 | 0.45 | 3.20 | |  |
| CAPTION: Cox model testing the association between adjuvant chemotherapy and survival, adjusted for clinically significant tumor factors and number of cycles of FOLFIRINOX: (A) in all patients, (B) in a landmark analysis starting at 3 months after surgery, (C) in a landmark analysis starting at 8 months after surgery, and (D) the same landmark analysis excluding all patients exceeding 4 cycles of neoadjuvant FOLFIRINOX. *Excluding 99 and ** excluding 20 patients with missing data on the number of adjuvant chemotherapy cycles. | | | | | | |
