# Supplementary material for: Surgical and Oncological Outcomes After Preoperative FOLFIRINOX Chemotherapy in Resected Pancreatic Cancer: An International Multicenter Cohort Study
Source: Ann Surg Oncol. 2022 Dec 20;30(3):1463–73. doi: 10.1245/s10434-022-12387-2 (PMC9908650; doi:10.1245/s10434-022-12387-2)
Supplement: Supplementary file 8 — (DOCX 16 KB) [file 10434_2022_12387_MOESM8_ESM.docx]

SUPPLEMENT S8. THE ASSOCIATION BETWEEN DURATION OF PREOPERATIVE THERAPY AND SURVIVAL.

|  | **All patients starting at time of diagnosis (n=423)** | | | | |
| --- | --- | --- | --- | --- | --- |
| **Covariate** | **HR** | **P-Value** | **LCI** | **UCI** |  |
| Age per year | 0.99 | 0.487 | 0.98 | 1.01 |  |
| BMI | 1.03 | 0.076 | 1.00 | 1.07 |  |
| Tumor differentiation per grade | 1.44 | 0.009 | 1.11 | 1.88 |  |
| Malignant lymph-node-ratio | 1.14 | 0.024 | 1.02 | 1.28 |  |
| Duration of preoperative therapy | 0.83 | 0.233 | 0.60 | 1.13 |  |
| Resection margin | 1.64 | 0.001 | 1.22 | 2.21 |  |

CAPTION: Cox model testing the association between duration of preoperative therapy (<5 months or ≥5 months preoperative therapy), adjusted for clinically significant tumor factors. Abbreviations: BMI, body mass index; HR, hazard ratio; LCI, lower 95% confidence interval; UCI, upper 95% confidence interval.
